# Adaptation and content validity of the Brazilian version of the Mental Health Literacy questionnaire

**DOI:** 10.1590/0034-7167-2024-0309

**Published:** 2025-12-08

**Authors:** Wanderson Carneiro Moreira, Luísa Campos, Pedro Dias, Maria do Perpétuo Socorro de Sousa Nóbrega

**Affiliations:** I Universidade de São Paulo. São Paulo, São Paulo, Brazil; II Universidade Católica Portuguesa. Porto, Portugal; III Universidade dos Açores. Ponta Delgada, Portugal

**Keywords:** Young Adult, Mental Health, Public Health, Mental Health Literacy, Validation Studies, Adulto Joven, Salud Mental, Salud Pública, Alfabetização en Salud Mental, Estudios de Validação

## Abstract

**Objectives:**

to perform cross-cultural adaptation and content validity of the Mental Health Literacy questionnaire into Brazilian Portuguese.

**Methods:**

a validity study developed according to the following stages: (1) assessment of conceptual and item equivalence; (2) idiomatic and semantic equivalence (translation, back-translation, formal assessment, critical analysis by 17 experts and pre-testing with 32 young adults, using the survey technique); (3) operational equivalence.

**Results:**

important changes were made to ensure instrument validity. The Content Validity Index for the Brazilian context was 97.2% (feasibility) and 98.9% (relevance). The instrument proved to be understandable and easy to apply in pre-testing.

**Final Considerations:**

the Mental Health Literacy questionnaire, Brazilian version, contributes to assessing mental health literacy as well as to improving the quality of actions to promote, prevent and protect mental health in young people.

## RESUMEN

**Objetivos:**

realizar la adaptação transcultural y validação de contenido del *Mental Health Literacy questionnaire* al portugués brasileño.

**Método:**

estudio de validação desarrollado en términos de los siguientes pasos: (1) evaluação de la equivalencia conceptual y de ítems; (2) equivalencia idiomática y semántica (traducação, retrotraducação, evaluação formal, análisis crítico por 17 expertos y prueba previa con 32 adultos jóvenes, utilizando la técnica de encuesta); (3) equivalencia operacional.

**Resultados:**

se realizaron cambios importantes para asegurar la validez del instrumento. El índice de validez de contenido para el contexto brasileño fue de 97,2% (viabilidad) y 98,9% (relevancia). El instrumento demostró ser comprensible y fácil de aplicar en la prueba previa.

**Consideraciones Finales:**

el *Mental Health Literacy questionnaire*, versião brasileña, contribuye a la evaluação de la alfabetização en salud mental, así como a la mejora de la calidad de las acciones de promoação, prevenação y protecação de la salud mental en jóvenes.

## INTRODUCTION

Mental health conditions represent a leading cause of disability, contributing substantially to the global burden of disease^(1,2)^. In this sense, public mental health evolves with the impact of global challenges, namely climate change, migration and health crises. Ensuring healthy lives and promoting well-being for all, at all ages, is the third of 17 Sustainable Development Goals (SDGs) in the United Nations (UN) 2030 Agenda. These issues require attention and priority focus in the development of strategies for mental health prevention, promotion and protection^(3)^.

One of these strategies could be mental health literacy, recognized as a determining factor in mental health and important in promoting mental health, with the potential to benefit individual and collective mental health, an essential resource for achieving economic, environmental and social ambitions regarding the SDGs^(4,5)^.

Mental health literacy is a concept introduced by Jorm *et al*.^(6)^ that was initially defined as knowledge and beliefs about mental disorders that aid in their recognition, management or prevention. An update of this concept included the ability to provide support to someone presenting with a mental health problem, i.e., first aid skills^(7)^. Thus, mental health literacy is not limited to having knowledge, as this is linked to beliefs that, together, determine attitudes such as resistance to seeking professional help.

Good levels of mental health literacy have a direct and positive impact on adulthood by enabling young people to acquire knowledge and define attitudes and behaviors that will accompany them in their future lives. In other words, mental health literacy provides this population with the ability to positively manage their thoughts and emotions to build healthy social and family relationships based on a strong and positive sense of identity^(3)^.

Without good levels of mental health literacy, and in the absence of the knowledge and skills necessary to prevent the development of mental disorders and promote good mental health, young people, as they reach adulthood, are more susceptible to mental illness and consequent chronicity^(7)^. Therefore, the transition between adolescence and adulthood represents an important time for priority intervention in the area of issues related to mental health literacy promotion.

Studies have shown the effectiveness of actions to promote mental health literacy in countries that have implemented it as a public health policy, such as Portugal and Australia. However, they warn that, for its implementation and success in other countries, it is necessary to assess mental health literacy in the groups of interest^(7,8)^.

Taking into account the evolution of the concept of mental health literacy and the limitations of previous measures (e.g., use of time-consuming vignettes, measures limited to specific mental health problems)^(8,9)^, Dias *et al*.^(10)^ developed a new instrument to provide a more up-to-date assessment of this construct, the Mental Health Literacy questionnaire (MHLq), version for young adults, which includes 29 items, assessed on a five-point Likert-type scale, organized into four dimensions: (1) knowledge of mental health problems; (2) erroneous beliefs/stereotypes; (3) help-seeking and first aid skills; and (4) self-help strategies. The preliminary study of the psychometric properties of this instrument showed adequate levels of validity and internal consistency^(10)^.

In a literature review, a considerable gap in knowledge was observed regarding mental health literacy assessment in the Americas region, especially in Brazil, which makes it difficult to establish a situational overview and propose public policies^(9)^. Furthermore, the use of instruments in other languages and contexts must be preceded by adaptation to the local culture and psychometric study. Although Portugal and Brazil share the same language, the Portuguese language spoken in Brazil presents some differences in terms of vocabulary, phonetics and syntax in relation to the Portuguese language spoken in Portugal.

## OBJECTIVES

To perform cross-cultural adaptation and content validity of MHLq into Brazilian Portuguese.

## METHODS

### Ethical and legal aspects

The study was approved by the Research Ethics Committee. All those involved in the study voluntarily agreed to participate and signed the Informed Consent Form (ICF) by means of electronic signature, in compliance with national and international standards for research involving human beings.

### Study design

This is a validity study that followed the recommendations for instrument validity proposed by Reichenheim and Moraes^(11)^ regarding the following stages: (1) assessment of conceptual and item equivalence; (2) idiomatic and semantic equivalence (translation, back-translation, formal assessment, critical analysis by an expert committee and pre-testing); and (3) operational equivalence.

### Setting and study participants

The study was conducted virtually in all regions of Brazil, between March and November 2020, with 53 participants: target population (32); expert committee (17); and translators (05).

### Study stages

A literature review was conducted to identify instruments that assess mental health literacy in adolescents and young people. The results indicated that MHLq had good conceptual equivalence regarding the definition of mental health and the potential for cross-cultural adaptation for Brazil. The analysis was based on the composition of the questionnaires, number of items, form of application and responses. The broadest possible spectrum of measurability of the construct was taken into consideration.

To carry out the item equivalence stage and subsequent stages, authorization was obtained from the authors of the original instrument. Then, a consultation was carried out on the *Lattes* Platform to identify professionals with expertise in the thematic area and/or method, in order to form the expert committee, following the classification parameters adapted in accordance with Fehring's proposal^(12)^, selecting those who achieved a minimum score of five points.

Twenty-two professionals from all geographic regions of the country were identified; of these, 17 agreed to participate in the study after a formal invitation sent via email, of which 11 (64.7%) were women; 14 were between 27 and 57 years of age (82.3%); 14 had master's and doctoral degrees (82.3%); were nurses, psychologists (2) and a physician (1); 15 had graduated from two to 31 years ago (88.2%); six worked in the areas of mental health and psychometry (35.3%); and four worked with youth (23.5%).

The expert committee analyzed each item of MHLq regarding the relevance of the quality dimension to be measured and its feasibility in the Brazilian context. The original instrument, a sociodemographic questionnaire and an assessment script adapted on a Likert scale from 1 to 4 points were made available to the committee via Google Docs^®^: 1 (irrelevant or unfeasible); 2 (not very relevant or not very feasible); 3 (relevant or feasible); and 4 (very relevant or very feasible)^(11,13)^.

To assess semantic equivalence, the original questionnaire was translated (T1 and T2) from Portuguese from Portugal to Portuguese from Brazil, independently, by two professionals: one with training in mental health and the other with training in linguistics, both fluent in Portuguese. These two versions were back-translated (BT1 and BT2) into Portuguese from Portugal by two other translators, native speakers of Portugal, who did not have access to the original version. The two back-translated versions were then assessed by a healthcare professional with knowledge of Brazilian and Portuguese culture, who first assessed semantic equivalence between the original questionnaire and each back-translation, from the perspective of referential meaning of constituent words, and then assessed the general meaning^(11,13)^.

In the form used to assess the referential meaning, the original version was compared with the back-translated versions (BT1 and BT2) with the aim of assessing literal (denotative) correspondence between them. The pairwise equivalence of assertions was judged continuously on a scale from 0% to 100% in increasing order of literal equivalence^(11,13)^. The second aspect assessed was the general (connotative) meaning of each item relative to the Brazilian version compared to the original. This correspondence transcends the literal meaning of words, also reporting more subtle aspects, such as meanings that certain terms may have in the target population's cultural context. A categorization into four levels was chosen: unchanged; slightly changed; greatly changed; or completely changed^(11,13)^.

After modifications were made at this stage, the version obtained was reviewed by the expert committee, aiming to obtain the version to be tested, also using a Likert-type scale of 1 to 4 points: 1 (irrelevant or unfeasible); 2 (not very relevant or not very feasible); 3 (relevant or feasible); and 4 (very relevant or very feasible)^(11,13)^.

For the pre-testing stage, also called cognitive debriefing^(13)^, following snowball sampling criteria^(14)^, 32 participants aged between 18 and 25 years were recruited for the study through digital social networks (Facebook^®^, Instagram^®^ and WhatsApp^®^), the majority of whom were men (19), living in the Southeast region (11) and had incomplete higher education (14). Tourists were excluded. To define the age range, Sposito and Tarábola^(15)^ classification was taken into consideration, which considers a young adult to be someone aged between 18 and 25 years, an age range corresponding to the target audience of the questionnaire to be adapted.

Participants initially signed the virtual ICF and answered a sociodemographic questionnaire that was made available online. A group was then created on the WhatsApp^®^ app to schedule remote interviews, a procedure that was necessary due to the current social distancing measures imposed by the pandemic. Participants were divided into three groups to participate in remote meetings via Google Meet^®^, which were scheduled in advance.

Pre-testing was conducted using the probing technique, which aims to avoid systematic errors in understanding. For each question, the interviewer offered respondents the opportunity to openly state what they understood about the question^(16,17)^, i.e., there was also the proposal of a structured technique, the Three-Step Test-Interview, proposed by Hak, Veer and Jansen^(18)^. In this stage, the researchers assessed the relevance and adequacy of the vehicle and format of questions/instructions, the administration setting, the application method, and the categorization method.

### Statistical analysis

The data were entered into a Microsoft Excel^®^ spreadsheet and processed using the Statistical Package for the Social Sciences version 22.0. A significance level of 5% was adopted for all tests used. For item and semantic equivalence, performed by the expert committee, the item-level Content Validity Index (I-CVI) was calculated by adding the agreement of items scored by the evaluators. Items that received scores of "1" or "2" were considered failed, being reviewed, modified and re-assessed, while items that received scores of "3" or "4" corresponded to approvals. Thus, I-CVI is defined as the ratio between the number of items that received scores of "3" or "4" and the number of evaluators^(19)^.

For the agreement rate among committee participants to be considered acceptable, an I-CVI was greater than 0.80^(20)^. To assess the complete questionnaire-level Content Validity Index (Q-CVI), arithmetic mean was estimated from I-CVI count. The approval parameter was at least 90%^(20)^.

## RESULTS

The cross-cultural adaptation process developed in this study lasted approximately ten months. MHLq, identified through a literature review, obtained items and composition considered pertinent by the evaluators who composed the expert committee, since all I-CVI exceeded the minimum approval standard of 80%, as well as Q-CVI, which exceeded 90%. However, some items initially rejected were assessed and modified for another round of assessment (Table 1).

**Table 1:** Content Validity Index regarding relevance and feasibility criteria of the Mental Health Literacy Questionnaire, Brazilian version, total and by item, São Paulo, São Paulo, Brazil, 2024.

MHLq-Br items	CVI (relevance)			CVI (feasibility)		
	Nº of failures (assessment 1)	Nº of failures (assessment 2)	I-CVI (%)	Nº of failures (assessment 1)	Nº of failures (assessment 2)	I-CVI (%)
1			100			100
2			100		-	95.2
3			100			100
4		-	95.2		-	95.2
5		-	90.5		-	85.7
6	9		100	7	-	95.2
7	6		100			100
8	9		100		-	90.5
9	6		100			100
10	17		100		-	81
11	5		100			100
12	7		100			100
13	14	-	90.5	7	-	95.2
14	6		100			100
15	12		100	7		100
16	8		100			100
17	6		100		-	95.2
18	10		100		-	95.2
19	11		100		-	95.2
20	6		100			100
21	12	-	90.5	6		100
22	7		100			100
23	14	-	95.2	6		100
24	6		100			100
25	9		100			100
26	5		100			100
27	7		100		-	95.2
28	7		100			100
29	7		100		-	90.5
30			100			100
31			100			100
32			100			100
33			100			100
Q-CVI	98.9			97.2		

MHLq-Br - Mental Health Literacy questionnaire, Brazilian version; I-CVI : item-level Content Validity Index.

In the initial translation into Brazilian Portuguese, two distinct translations were obtained, which were compared by the expert committee, and through consensus, a single version was constructed. In the back-translation stage, the summary version in Portuguese was back-translated into the original language by two native translators independently. The two back-translated versions were sent to a third native translator from the Portuguese country, who carried out a formal assessment of equivalence regarding the general and referential meanings. Overall, good semantic equivalence was evident. Concerning referential meaning, similarity was observed between the two back-translations and the original instrument. Considering the general meaning, translation 1 was the best assessed, receiving a rating of "unchanged" in 24 items (Table 2).

**Table 2:** Assessment of semantic equivalence by assessing referential and general equivalence between back-translated items and the original Mental Health Literacy questionnaire, São Paulo, São Paulo, Brazil, 2024.

	Evaluator's judgement Version 1 (T1+BT1) Nº of items (%)	Version 2 (T2 +BT2) Nº of items (%)
**Referential meaning (original/back-translation)**	90-100%	28 (86)	22 (76)
	70 < 90%	1 (14)	7 (24)
	50 < 70%	-	-
	< 50%	-	-
Total	29 (100)	29 (100)
**General meaning (original/translation)**	Unchanged	24 (41)	15 (41)
	Slightly changed	5 (10)	12
	Changed		2 (21)
	Greatly changed		
	Completely changed		
	Total	29 (100)	29 (100)

T1 : translation 1; T2 : translation 2; BT1 : back-translation 1; BT2 : back-translation 2.

Once the summary version was in hand, it was sent for assessment by the expert committee, which assigned scores of "1" and "2" to 24 items of the instrument and suggested modifications. The suggestions and changes were as follows: the items that contained the expression "*perturbação mental*" (mental disturbance) were changed to "*transtornos mentais*" (mental disorders), following the proposal of the Diagnostic and Statistical Manual of Mental Disorders, fifth edition, used most frequently in the Brazilian context. In items 1, 7 and 26, the committee suggested that the statement "*boa saúde mental*" (good mental health) be changed to "*manutenação da saúde mental*" (mental health maintenance). However, in agreement with the authors of MHLq, it was decided to maintain the original statement, as such replacement would not indicate a sense of improvement, but rather of stagnation.

For item 2, it was suggested that the term "*muito*" (very) be removed, but, in consensus between the researchers and the authors of MHLq, it was decided not to change this item, since it is not intended to measure intensity.

Some experts suggested that item 8 be changed, with the term "*psicólogo*" (psychologist) being replaced by "*enfermeiro*" (nurse). This request was not accepted by the original authors of MHLq. However, two items related to nursing professionals were added.

The term "*altera*" (changes), present in items 6, 13, 25, was changed to "*afeta*" (affects), as suggested by the committee, in order to maintain the wording in accordance with the original instrument. The same conduct was maintained in relation to items 20 and 23. Items 10, 13, 15 and 23 were rejected by the expert committee in the first assessment, requesting their exclusion. However, this suggestion was not accepted by the authors of MHLq, since these items refer to erroneous statements, with the aim of assessing stereotypes, considered factors that interfere with the levels of mental health literacy. After the committee understood the meaning of items, they were approved in the second assessment.

The other changes were related to terms or expressions for which minor modifications were made, in order to enable a better understanding by the target population. The final version was forwarded to the authors of MHLq, who appreciated it favorably. Pre-testing was carried out with 32 adolescents and young people (divided into three groups), through an online interview. Regarding operational equivalence, it was observed that, through the technique used, there were no difficulties for the sample members, proving to be understandable for the local reality. Response time varied from 15 to 30 minutes.

## DISCUSSION

Due to the low cost and ease of application of questionnaires for measuring mental health literacy in young people, and also due to the lack of Brazilian instruments aimed at assessing this latent variable, it was identified as important to carry out this study. The methodological procedures for cross-cultural adaptation used were effective and satisfactory, since content validity was analyzed by an expert committee and the application of the Brazilian Portuguese version to a sample of the target population through pre-testing.

The first step of the chosen method^(10)^ indicates the conduct of a review study. This step was essential for researchers to conduct a discussion on concepts of mental health^(21)^ and mental health literacy^(4)^, which endorsed the choice of MHLq as the most viable for cross-cultural adaptation for Brazil, due to the presentation format, application and proximity of the construct to the Brazilian reality, enabling the achievement of conceptual equivalence. Furthermore, evidence suggests that most available instruments for assessing mental health literacy assess specific mental health problems or diagnoses (e.g., schizophrenia, depression, substance use). However, MHLq fills these gaps by proposing an assessment from a comprehensive perspective of the construct, rather than focusing on a restricted number of mental disorders or specific dimensions of these disorders^(22)^.

Concerning the stages of idiomatic and semantic equivalence of items, it was found that the initial translation process of the original instrument, carried out by Portuguese speaking (semantics) professionals, was important for adapting the terms. It is important to consider the profile of translators to obtain good conceptual, item and semantic equivalence between the original and translated versions^(23)^.

In the idiomatic and semantic equivalence stage, another aspect worth highlighting is that, in back-translation, in addition to translators being bilingual and having training in the areas of languages and linguistics, linguistics and health, this stage was carried out blindly in relation to the initial translation stage. In addition to obtaining the back-translated versions and reaching consensus between both parties, there was a need for assessment by a third professional with knowledge of Portuguese from Portugal and Brazil and also with knowledge in the area of study of the instrument, an aspect highlighted in literature^(10)^. Furthermore, the back-translation stage is considered a content validity assessment process, as it analyzes whether the translated version accurately reflects the original instrument content.

The multidisciplinary composition of the expert committee, which included healthcare professionals, researchers in the areas of mental health, youth and psychometrics, one of the initial translators and a foreign-born member who was bilingual at the beginning of the study, was fundamental to the cross-cultural adaptation process.

Pre-testing, understood as the last stage of the cross-cultural adaptation process, whose objective is to analyze the understanding of the target population and the applicability of the instrument to the local reality, is common in the literature^(24,25)^. This stage was also considered an analysis of the content validity of the adapted instrument, as it allowed researchers to confirm whether it was applicable to the other reality.

Previously^(26)^, an assessment instrument was used at the end of the interview. In the present study, cognitive debriefing was chosen^(27)^. For each question, the respondent was given the opportunity to openly state what they understood about the question, in order to avoid systematic errors of understanding, which, when detected, were adjusted with the expert committee, an essential strategy to overcome limitations imposed by the virtual environment.

The results of this study were positive, since conceptual and item equivalences were relevant (98.9%) and viable (97.2%) for the Brazilian context, given that CVI values had results higher than the approval parameters indicated in literature^(19,20)^. Semantic, idiomatic and operational equivalences were essential for adapting the instrument to the Brazilian reality. The items proved to be easy to understand by the target population.

### Study limitations

It is important to note that the data were collected during the COVID-19 pandemic, which may have impacted the results, since awareness of mental health issues has generally increased during this period. Due to this fact, the pre-test was carried out entirely virtually, and to manage online platforms, a minimum level of literacy is required, an aspect that may have excluded participants with low educational levels, in addition to the condition of access to digital technologies.

### Contributions to nursing, health and public policies

The results of this study are timely and contribute to meeting the UN SDGs, especially topics 3 and 4^(27)^. Regarding the Brazilian reality, they will support actions and strategies that contribute to strengthening societies with higher levels of mental health literacy, as well as to advancing public policies, especially those aimed at adolescents and young people^(28)^. Furthermore, it will be a tool available for epidemiological research on mental health in Brazil, allowing comparability between national and international studies, contributing to scientific advancement related to mental health problems in the population.

Nurses, who are familiar with adolescence as a transition process, play an essential role in analyzing all the determinants of health literacy and in developing intervention programs aimed at preventing and promoting mental health in young people. These professionals have the opportunity to lead multidisciplinary teams in the use of these programs. Thus, they will be able to use and guide the use of this instrument in primary and/or specialized mental healthcare teams, constituting a tool for supporting and qualifying work in the public health field^(29-34)^.

### FINAL CONSIDERATIONS

The adaptation and validity of MHLq content for Brazil required significant adjustments to ensure item equivalence with MHLq. MHLq presents conceptual equivalence because it involves the construct of interest in its different dimensions. It presents semantic equivalence according to the assessment process of connotative and referential meaning. MHLq achieved operational equivalence due to its presentation and application format. The Brazilian version also requires compliance with the measurement equivalence assessment stages, such as reliability and validity of the instrument's dimensional structure.

## Data Availability

The research data are available only upon request.

## References

[1] (2022). Global, regional, and national burden of 12 mental disorders in 204 countries and territories, 1990:2019: a systematic analysis for the Global Burden of Disease Study 2019. Lancet Psychiatry..

[2] Wu Y, Wang L, Tao M, Cao H, Yuan H, Ye M (2023). Changing trends in the global burden of mental disorders from 1990 to 2019 and predicted levels in 25 years. Epidemiol Psychiatr Sci..

[3] Tullius J, Beukema L (2021). The importance of mental health literacy in times of crisis: adolescent mental health during the COVID-19 pandemic. Eur J Public Health..

[4] Jorm AF, Korten AE, Jacomb PA, Christensen H, Rodgers B, Pollitt P (1997). "Mental health literacy": a survey of the public's ability to recognise mental disorders and their beliefs about the effectiveness of treatment. Med J Aust..

[5] Jorm AF (2012). Mental health literacy: empowering the community to take action for better mental health. Am Psychol..

[6] Tully LA, Hawes DJ, Doyle FL, Sawyer MG, Dadds MR (2019). A national child mental health literacy initiative is needed to reduce childhood mental health disorders. Aust N Z J Psychiatry..

[7] Nobre J, Oliveira AP, Monteiro F, Sequeira C, Ferré-Grau C (2021). Promotion of mental health literacy in adolescents: a scoping review. Int J Environ Res Public Health..

[8] Marinucci A, Grové C, Allen KA (2021). A scoping review and analysis of mental health literacy interventions for children and youth. Sch Psychol Rev..

[9] Mansfield R, Patalay P, Humphrey N (2020). A systematic literature review of existing conceptualisation and measurement of mental health literacy in adolescent research: current challenges and inconsistencies. BMC Public Health..

[10] Dias P, Campos L, Almeida H, Palha F (2018 Jun). Mental health literacy in young adults: adaptation and psychometric properties of the mental health literacy questionnaire. Int J Environ Res Public Health.

[11] Reichenheim ME, Moraes CL (2007). Operacionalização de adaptação transcultural de instrumentos de aferiação usados em epidemiologia. Rev Saúde Pública..

[12] Fehring RJ (1987). Methods to validate nursing diagnoses. Nurs Fac Res Publ..

[13] Moraes CL, Hasselmann MH, Reichenheim ME Adaptação transcultural para o português do instrumento "Revised Conflict Tactics Scales (CTS2)" utilizado para identificar violência entre casais. Cad Saúde Pública [Internet].

[14] Biernacki P, Walford D (1981). Snowball sampling: problems and techniques of chain referral sampling. Sociol Methods Res..

[15] Sposito MP, Tarábola FS (2017). Entre luzes e sombras: o passado imediato e o futuro possível da pesquisa em juventude no Brasil. Rev Bras Educ..

[16] Herdman M, Fox-Rushby J, Badia X (1998). A model of equivalence in the cultural adaptation of HRQoL instruments: the universalist approach. Qual Life Res..

[17] Guillemin F, Bombardier C, Beaton D (1993). Cross-cultural adaptation of health-related quality of life measures: literature review and proposed guidelines. J Clin Epidemiol..

[18] Hak T, Veer KVD, Jansen H (2008). The Three-Step Test-Interview (TSTI): an observation-based method for pretesting self-completion questionnaires. Surv Res Methods..

[19] Wynd CA, Schmidt B, Schaefer MA (2003). Two quantitative approaches for estimating content validity. West J Nurs Res..

[20] Polit DF, Beck CT (2006). The content validity index: are you sure you know what's being reported? Critique and recommendations. Res Nurs Health.

[21] World Health Organization (2013). Mental health action plan 2013:2020. [Internet].

[22] Spiker DA, Hammer JH (2019). Mental health literacy as theory: current challenges and future directions. J Ment Health..

[23] Moraes KL, Brasil VV, Mialhe FL, Sampaio HAC, Sousa ALL, Canhestro MR (2021). Validação do Health Literacy Questionnaire (HLQ) para o português brasileiro. Acta Paul Enferm..

[24] Sousa KHJF, Lluch-Canut MT, Gallasch CH, Zeitoune RCG (2021). Adaptação transcultural do Cuestionario de Salud Mental Positiva para estudantes de enfermagem no contexto brasileiro. Texto Contexto Enferm..

[25] Zanardo ABR, Ventura CAA (2022). Adaptação cultural e validação do módulo Strategies to end seclusion restraint do ToolKit QualityRights. Rev Latino-Am Enferm..

[26] Weissheimer AM (2007). Traduação, adaptação transcultural e validação para uso no Brasil do instrumento Prenatal Psychosocial Profile [Tese].

[27] Paskulin LMG, Aires M, Valer DB, Morais EP, Freitas IBA (2011). Adaptação de um instrumento que avalia alfabetização em saúde das pessoas idosas. Acta Paul Enferm..

[28] Roma JC (2019). Os objetivos de desenvolvimento do milênio e sua transiação para os objetivos de desenvolvimento sustentável. Cienc Cult..

[29] Olivari C, Casañas R (2020). Relevancia de la alfabetização en salud mental para adolescentes en tiempos del covid-19. Cuad Neuropsicol..

[30] Tay JL, Tay IF, Klainin-Yobas P (2018). Mental health literacy levels. Arch Psychiatr Nurs..

[31] Gonçalves A, Cabral L, Cruz C, Chaves C, Sequeira C, Rodrigues JF (2021). Literacia em saúde mental positiva nos enfermeiros de cuidados de saúde primários. Int J Dev Educ Psychol..

[32] Costa T, Sampaio F, Sequeira C, Lluch-Canut T, Moreno-Poyato AR (2021). A qualitative study exploring adolescents' perspective about mental health first aid training programmes promoted by nurses in upper secondary schools. Int J Ment Health Nurs..

[33] Pinto DL, Parisod H, Nyman J, Barroso TMMDA (2022). Efetividade da versão portuguesa do Fume na literacia em saúde de adolescentes acerca do tabaco. Rev Latino-Am Enferm..

[34] Moreira WC, Sousa AR, Cardoso RSS, Queiroz AM, Oliveira MAF, Sequeira CACJ (2022). COVID-19 in Brazil: Are there differences in Mental Health Literacy between young and aged men?. Rev Latino-Am Enfermagem..

