# Supplementary material for: Adaptation and content validity of the Brazilian version of the Mental Health Literacy questionnaire
Source: Rev Bras Enferm. 2025 Dec 8;78(4):e20240309. doi: 10.1590/0034-7167-2024-0309 (PMC12716808; doi:10.1590/0034-7167-2024-0309)
Supplement: Translation

# Translation: Adaptação e validação de conteúdo da versão brasileira do Mental Health Literacy questionnaire

**DOI:** 10.1590/0034-7167-2024-0309pt

**Authors:** Wanderson Carneiro Moreira, Luísa Campos, Pedro Dias, Maria do Perpétuo Socorro de Sousa Nóbrega

**Affiliations:** I Universidade de São Paulo. São Paulo, São Paulo, Brazil; II Universidade Católica Portuguesa. Porto, Portugal; III Universidade dos Açores. Ponta Delgada, Portugal

**Keywords:** Jovem Adulto, Saúde Mental, Saúde Pública, Letramento em Saúde, Estudos de Validação

## Abstract

**Objetivos:**

realizar a adaptação transcultural e validação de conteúdo do *Mental Health Literacy questionnaire* para o português brasileiro.

**Métodos:**

estudo de validação desenvolvido quanto à s etapas: (1) avaliação da equivalência conceitual e de itens; (2) equivalência idiomática e semântica (traduação, retrotraduação, apreciação formal, análise crítica por 17 especialistas e pré-teste com 32 jovens adultos, a partir da técnica de sondagem); (3) equivalência operacional.

**Resultados:**

alterações importantes foram realizadas para garantir a validade do instrumento. O Indice de Validade de Conteúdo referente ao contexto brasileiro foi de 97,2% (viabilidade) e 98,9% (relevância). O instrumento mostrou-se compreensível e de fácil aplicação no pré-teste.

**Considerações Finais:**

o *Mental Health Literacy questionnaire*, versão brasileira, contribui para avaliação do letramento em saúde mental, bem como para a melhoria da qualidade das ações de promoação, prevenação e proteação da saúde mental em idades jovens.

## INTRODUÇÃO

As condições de saúde mental representam uma das principais causas de incapacidade, contribuindo substancialmente para a carga global de doença^(1,2)^. Nesse sentido, a saúde mental pública evolui com o impacto de desafios globais, nomeadamente mudanças climáticas, migração e crises de saúde. Assegurar uma vida saudável e promover o bem-estar para todos, em todas as idades, é o terceiro de 17 Objetivos de Desenvolvimento Sustentável (ODS) na Agenda 2030 da Organização das Nações Unidas (ONU). Tais questões necessitam de atenação e foco prioritário na elaboração de estratégias de prevenação, promoação e proteação da saúde mental^(3)^.

Uma dessas estratégias pode ser o letramento em saúde mental, reconhecido como fator determinante de saúde mental e importante na promoação da saúde mental, com potencialidade de beneficiar a saúde mental individual e coletiva, recurso essencial para alcançar as ambições econômicas, ambientais e sociais quanto aos ODS^(4,5)^.

O letramento em saúde mental é um conceito introduzido por Jorm *et al*.^(6)^ que foi inicialmente definido como conhecimentos e crenças sobre os transtornos mentais que auxiliam no seu reconhecimento, gestão ou prevenação. Uma atualização desse conceito incluiu a capacidade de fornecer suporte a alguém que apresenta um problema de saúde mental, ou seja, habilidades de primeiros socorros^(7)^. Nesse sentido, o letramento em saúde mental não se limita a ter conhecimento, pois este está ligado a crenças que, em conjunto, determinam atitudes como, por exemplo, a resistência em procurar ajuda profissional.

Bons níveis de letramento em saúde mental geram impacto direto e positivo na vida adulta ao permitir que jovens adquiram conhecimento e definam atitudes e comportamentos que os acompanharão em suas vidas futuras. Em outras palavras, o letramento em saúde mental promove a esta população a capacidade de gerenciar positivamente seus pensamentos e emoções para construir relacionamentos sociais e familiares saudáveis baseados em um forte e positivo senso de identidade^(3)^.

Sem bons níveis de letramento em saúde mental, e na ausência de conhecimento e habilidades necessárias para prevenir o desenvolvimento de transtornos mentais e promover uma boa saúde mental, os jovens, à medida que atingem a idade adulta, são mais suscetíveis ao adoecimento mental e consequente cronificação^(7)^. Por isso, a transiação entre a adolescência e a idade adulta representa um momento importante para intervenação prioritária no à ¢mbito das questões ligadas à promoação do letramento em saúde mental.

Estudos têm indicado a eficácia de ações de promoação do letramento em saúde mental em países que o implantaram enquanto política pública de saúde, a exemplo de Portugal e Austrália. No entanto, alertam que, para sua implantação e à ªxito em outros países, é necessária avaliação do letramento em saúde mental nos grupos de interesse^(7,8)^.

Levando em consideração a evoluação do conceito de letramento em saúde mental e as limitações de medidas anteriores (por exemplo, uso de vinhetas demoradas, medidas limitadas a problemas específicos de saúde mental)^(8,9)^, Dias *et al*.^(10)^ desenvolveram um novo instrumento para fornecer uma avaliação mais atualizada deste construto, o *Mental Health Literacy questionnaire* (MHLq), questionário de Literacia em Saúde Mental, versão para jovens adultos, que inclui 29 itens, avaliados em uma escala do tipo Likert de cinco pontos, organizados em quatro dimensões: (1) conhecimento sobre problemas de saúde mental; (2) crenças/estereótipos errôneos; (3) habilidades em primeiros socorros e busca de ajuda; e (4) estratégias de autoajuda. O estudo preliminar das propriedades psicométricas deste instrumento apresentou níveis adequados de validade e consistência interna^(10)^.

Em revisão de literatura, observou-se uma considerável lacuna do conhecimento sobre a avaliação do letramento em saúde mental na região das Américas, sobretudo no Brasil, o que dificulta estabelecer um panorama situacional e propor políticas públicas^(9)^. Ademais, a utilização de instrumentos em outros idiomas e contextos deve ser precedida de adaptação para a cultura local e estudo psicométrico. Embora Portugal e Brasil compartilhem a mesma língua, a língua portuguesa falada no Brasil apresenta algumas diferenças em termos de vocabulário, fonética e sintaxe em relação à língua portuguesa falada no país luso.

## OBJETIVOS

Realizar a adaptação transcultural e validação de conteúdo do MHLq para o português brasileiro.

## MÃ‰TODOS

### Aspectos éticos e legais

O estudo foi aprovado pelo Comitê de Ética em Pesquisa. Todos os envolvidos no estudo aceitaram participar voluntariamente e assinaram o Termo de Consentimento Livre e Esclarecido (TCLE), por meio de assinatura eletrônica, atendendo à s normas nacionais e internacionais de pesquisa envolvendo seres humanos.

### Tipo de estudo

Estudo de validação que seguiu as recomendações de validação de instrumentos propostas por Reichenheim e Moraes^(11)^ quanto à s seguintes etapas: (1) avaliação da equivalência conceitual e de itens; (2) equivalência idiomática e semântica (traduação, retrotraduação, apreciação formal, análise crítica por um comitê de especialista e pré-teste); e (3) equivalência operacional.

### Cenário e participantes do estudo

O estudo foi conduzido virtualmente em todas as regiões do Brasil, entre março e novembro de 2020, com 53 participantes: população-alvo (32); comitê de especialistas (17); e tradutores (05).

### Etapas do estudo

Realizou-se revisão da literatura para identificar instrumentos que avaliam o letramento em saúde mental em adolescentes e jovens. Os resultados apontaram o MHLq com boa equivalência conceitual quanto à definiação de saúde mental e à potencialidade de adaptação transcultural para o Brasil. A análise baseou-se na composiação dos questionários, quantidade de itens, forma de aplicação e respostas. Levou-se em consideração o maior espectro possível de mensurabilidade do construto.

Para a realização da etapa de equivalência de itens e subsequentes, obteve-se autorização dos autores do instrumento original. Em seguida, realizou-se consulta na Plataforma *Lattes* para identificar profissionais com *expertise* na área temática e/ou método, a fim de formar o comitê de especialistas, seguindo os parâmetros da classificação adaptado em conformidade com a proposta de Fehring^(12)^, sendo selecionados os que atingirem pontuação mínima de cinco pontos.

Identificaram-se 22 profissionais de todas as regiões geográficas do país; destes, 17 aceitaram participar do estudo após convite formal enviado via *e-mail*, dos quais 11 (64,7%) eram mulheres; 14 estavam na faixa etária de 27 a 57 anos (82,3%); 14 possuíam mestrado e doutorado (82,3%); eram enfermeiros(as), psicólogos (2) e médico (1); 15 tinham tempo de formação de dois a 31 anos (88,2%); seis atuavam nas áreas da saúde mental e psicometria (35,3%); e quatro atuavam com juventudes (23,5%).

O comitê de especialistas realizou a análise de cada item do MHLq quanto à relevância da dimensão da qualidade que se pretende aferir e à viabilidade ao contexto brasileiro. Foram disponibilizados ao comitê via *Google Docs* ^®^ o instrumento original, um questionário sociodemográfico e um roteiro de avaliação adaptado em escala do tipo Likert de 1 a 4 pontos: 1 (irrelevante ou inviável); 2 (pouco relevante ou pouco viável); 3 (relevante ou viável); e 4 (muito relevante ou muito viável)^(11,13)^.

Para avaliação da equivalência semântica, o questionário original foi traduzido (T1 e T2) do português de Portugal para o português do Brasil, de modo independente, por duas profissionais: uma com formação na área da saúde mental e outra com formação na área da linguística, ambas fluentes na língua portuguesa. Essas duas versões foram retrotraduzidas (RT1 e RT2) para o português de Portugal por outros dois tradutores, nativos de Portugal, que não tiveram acesso à versão original. Em seguida, as duas versões retrotraduzidas foram apreciadas por um profissional de saúde e conhecedor da cultura brasileira e portuguesa que, primeiro, avaliou a equivalência semântica entre o questionário original e cada uma das retrotraduções, sob a perspectiva do significado referencial das palavras constituintes, e, em seguida, apreciou o significado geral^(11,13)^.

No formulário utilizado para apreciar o significado referencial, a versão original foi comparada com as retrotraduzidas (RT1 e RT2), com o objetivo de avaliar a correspondência literal (denotativa) entre elas. A equivalência entre pares das assertivas foi julgada de forma contínua em uma escala de 0% a 100% por ordem crescente de equivalência literal^(11,13)^. O segundo aspecto apreciado foi o significado geral (conotativo) de cada item relativo à versão brasileira em comparação ao original. Essa correspondência transcende a literalidade das palavras, reportando também aspectos mais sutis, como as significações que determinados termos podem apresentar no contexto cultural da população-alvo. Optou-se por uma categorização em quatro níveis: inalterado; pouco alterado; muito alterado; ou completamente alterado^(11,13)^.

Após modificações realizadas nesta fase, a versão obtida foi revisada pelo comitê de especialistas, visando à obtenação da versão a ser testada, também por meio de escala do tipo Likert de 1 a 4 pontos: 1 (irrelevante ou inviável); 2 (pouco relevante ou pouco viável); 3 (relevante ou viável); e 4 (muito relevante ou muito viável)^(11,13)^.

Para a etapa de pré-teste, também denominada de interrogatório cognitivo (*cognitive debriefing*)^(13)^, seguindo os critérios da amostragem bola de neve^(14)^, foram recrutados para o estudo, por meio de redes sociais digitais (*Facebook* ^®^, *Instagram* ^®^ *e WhatsApp* ^®^), 32 participantes, entre 18 e 25 anos de idade, sendo a maioria homens (19), residentes na região Sudeste (11) e nível de escolaridade superior incompleto (14). Turistas foram excluídos. Para definiação da faixa etária, levou-se em consideração a classificação de Sposito e Tarábola^(15)^, que considera jovem adulto aquele com idade entre 18 e 25 anos, faixa etária correspondente à do público-alvo do questionário a ser adaptado.

Os participantes, inicialmente, assinaram o TCLE virtual e responderam a um questionário sociodemográfico virtualmente disponibilizado. Em seguida, criou-se um grupo no aplicativo *WhatsApp* ^®^ para agendamento das entrevistas remotas, procedimento necessário devido ao momento de distanciamento social em decorrência da pandemia. Os participantes foram divididos em três grupos para participação nos encontros remotos via *Google Meet* ^®^, os quais foram previamente agendados.

O pré-teste foi realizado a partir da técnica de sondagem, que objetiva evitar erros sistemáticos de compreensão. A cada pergunta, o entrevistador ofereceu a oportunidade aos respondentes de dizer abertamente o que entenderam sobre a pergunta^(16,17)^, ou seja, houve também a proposta de técnica estruturada, o Teste de Entrevista em Três Passos, proposto por Hak, Veer e Jansen^(18)^. Nessa etapa, os pesquisadores avaliaram a pertinência e adequação do veículo e formato das questões/instruções, o cenário de administração, o modo de aplicação, e o modo de categorização.

### Análise estatística

Os dados foram inseridos em planilha do programa *Microsoft Excel* ^®^ e processados no *software Statistical Package for the Social Sciences*, versão 22.0. Para todos os testes utilizados, foi adotado um nível de significância de 5%.

Para a equivalência de itens e semântica, realizada pelo comitê de especialistas, foi calculado o Índice de Validade de Conteúdo por item (IVC-I) mediante a soma de concordância dos itens pontuados pelos avaliadores. Os itens que receberam pontuações "1" ou "2" foram considerados reprovados, sendo revisados, modificados e reavaliados, enquanto que os itens que receberam pontuações "3" ou "4" corresponderam à s aprovações. Dessa forma, o IVC-I é definido como a razão entre o número de itens que receberam pontuações "3" ou "4" pelo número de avaliadores^(19)^.

Para que a taxa de concordância entre os participantes do comitê fosse considerada aceitável, o IVC-I foi superior a 0,80^(20)^. Para avaliar o Índice de Validade de Conteúdo do questionário completo (IVC-Q), foi estimada a média aritmética a partir da contagem dos IVC-I. O parâmetro de aprovação foi de no mínimo 90%^(20)^.

## RESULTADOS

O processo de adaptação transcultural desenvolvido neste estudo durou aproximadamente dez meses. O MHLq, identificado por meio de revisão de literatura, obteve itens e composiação considerados pertinentes perante os avaliadores que compuseram o comitê de especialistas, uma vez que todos os IVC-I superaram o padrão mínimo de aprovação de 80%, bem como o IVC-Q, que ultrapassou 90%. Todavia, alguns itens inicialmente reprovados foram apreciados e modificados para outra rodada de avaliação (Tabela 1).

**Tabela 1:** Índice de Validade de Conteúdo referente aos critérios de relevância e viabilidade do *Mental Health Literacy questionnaire*, versão brasileira, total e por item, São Paulo, São Paulo, Brasil, 2024.

Itens do MHLq-Br	IVC (relevância)			IVC (viabilidade)		
	Nº de reprovações (avaliação 1)	Nº de reprovações (avaliação 2)	IVC-I (%)	Nº de reprovações (avaliação 1)	Nº de reprovações (avaliação 2)	IVC-I (%)
1			100			100
2			100		-	95,2
3			100			100
4		-	95,2		-	95,2
5		-	90,5		-	85,7
6	9		100	7	-	95,2
7	6		100			100
8	9		100		-	90,5
9	6		100			100
10	17		100		-	81
11	5		100			100
12	7		100			100
13	14	-	90,5	7	-	95,2
14	6		100			100
15	12		100	7		100
16	8		100			100
17	6		100		-	95,2
18	10		100		-	95,2
19	11		100		-	95,2
20	6		100			100
21	12	-	90,5	6		100
22	7		100			100
23	14	-	95,2	6		100
24	6		100			100
25	9		100			100
26	5		100			100
27	7		100		-	95,2
28	7		100			100
29	7		100		-	90,5
30			100			100
31			100			100
32			100			100
33			100			100
IVC-Q	98,9			97,2		

MHLq-Br - Mental Health Literacy questionnaire, versão brasileira; IVC-I - Índice de Validade de Conteúdo por item.

Na traduação inicial para o português brasileiro, obtiveram-se duas traduções distintas, que foram comparadas pelo comitê de especialistas, e mediante consenso, foi construída uma única versão. Na etapa de retrotraduação, a versão síntese em português foi retrotraduzida para a língua original por dois tradutores nativos de forma independente. As duas versões retrotraduzidas foram encaminhadas para uma terceira tradutora nativa do país luso, que realizou uma apreciação formal da equivalência referente aos significados geral e referencial. No geral, evidenciou-se uma boa equivalência semântica. Quanto ao significado referencial, observou-se similaridade entre as duas retrotraduções e o instrumento original. Considerando o significado geral, a traduação 1 foi a melhor avaliada, recebendo classificação "inalterado" em 24 itens (Tabela 2).

**Tabela 2:** Avaliação da equivalência semântica por meio da apreciação da equivalência referencial e geral entre os itens das retrotraduções e o *Mental Health Literacy questionnaire* original, São Paulo, São Paulo, Brasil, 2024.

	Julgamento do avaliador	Versão 1 (T1+RT1) Nº de itens (%)	Versão 2 (T2 +RT2) Nº de itens (%)
**Significado referencial (original/retrotraduação)**	90-100%	28 (86)	22 (76)
	70 < 90%	1 (14)	7 (24)
	50 < 70%	-	-
	< 50%	-	-
	Total	29 (100)	29 (100)
**Significado geral (original/traduação)**	Inalterado	24 (41)	15 (41)
	Pouco alterado	5 (10)	12
	Alterado		2 (21)
	Muito alterado		
	Completamente alterado		
	Total	29 (100)	29 (100)

T1 : traduação 1; T2 : traduação 2; RT1 : retrotraduação 1; RT2 : retrotraduação 2.

De posse da versão síntese, encaminhou-se para a avaliação pelo comitê de especialistas, que atribuiu pontuações "1" e "2" para 24 itens do instrumento e sugeriram modificações. As sugestões e alterações foram as seguintes: os itens que continham a expressão "perturbação mental" foram alterados para "transtornos mentais", seguindo a proposiação da 5Âª ediação do Manual de Diagnóstico e Estatístico de Transtornos Mentais, utilizado com maior frequência no contexto brasileiro. Nos itens 1, 7 e 26, o comitê sugeriu que o enunciado "boa saúde mental" fosse alterado para "manutenação da saúde mental". No entanto, em consenso com os autores do MHLq, optou-se por manter o enunciado original, pois a substituiação não evidenciaria sentido de melhora, mas de estagnação.

Para o item 2, foi sugerida a retirada do termo "muito", porém, em consenso entre os pesquisadores e os autores do MHLq, optou-se por não alterar o item, pois o mesmo não tem finalidade de medir intensidade.

Alguns especialistas sugeriram que o item 8 fosse alterado, com a substituiação do termo "psicólogo" por "enfermeiro". Essa solicitação não foi acatada pelos autores originais do MHLq. No entanto, foram acrescentados dois itens relacionados ao profissional enfermeiro.

O termo "altera", presente nos itens 6, 13, 25, foi modificado para "afeta", conforme sugestão do comitê para manter o enunciado de acordo com o instrumento original. A mesma conduta foi mantida em relação aos itens 20 e 23. Os itens 10, 13, 15 e 23 foram reprovados pelo comitê de especialistas na primeira avaliação, solicitando a exclusão destes. No entanto, tal sugestão não foi acatada pelos autores do MHLq, pois esses itens referem-se a afirmações errôneas, no intuito de avaliar estereótipos, considerados fatores que interferem nos níveis de letramento em saúde mental. Após o comitê entender o sentido dos itens, estes foram aprovados na segunda avaliação.

As demais alterações foram referentes a termos ou expressões para os quais foram realizadas pequenas modificações, no intuito de possibilitar uma melhor compreensão da população-alvo. A versão final foi encaminhada para os autores do MHLq, que o apreciou favoravelmente.

O pré-teste foi realizado com 32 adolescentes e jovens (divididos em três grupos), por meio de entrevista *online*. Quanto à equivalência operacional, observou-se que, por meio da técnica utilizada, não houve dificuldades para os integrantes da amostra, mostrando-se compreensível para a realidade local. O tempo de resposta variou de 15 a 30 minutos.

## DISCUSSÃƒO

Devido à s características de baixo custo e facilidade de aplicação de questionários para a aferiação do letramento em saúde mental em idades jovens, e ainda pela lacuna de instrumentos brasileiros voltados para a avaliação dessa variável latente, identificou-se como importante a realização deste estudo. Os procedimentos metodológicos de adaptação transcultural utilizados foram eficazes e satisfatórios, uma vez que a validade de conteúdo foi analisada por meio de um comitê de especialistas e da aplicação da versão em português brasileiro a uma amostra da população-alvo por meio de pré-teste.

O primeiro passo do método escolhido^(10)^ indica a conduação de estudo de revisão. Esse passo foi essencial para que os pesquisadores realizassem uma discussão sobre os conceitos de saúde mental^(21)^ e letramento em saúde mental^(4)^, que endossou a escolha do MHLq como o mais viável para a adaptação transcultural para o Brasil, devido ao formato de apresentação, aplicação e proximidade do construto com a realidade brasileira, possibilitando a realização e alcance da equivalência conceitual. Além disso, evidências apontam que a maioria dos instrumentos disponíveis para avaliação do letramento em saúde mental avalia problemas ou diagnósticos específicos de saúde mental (por exemplo, esquizofrenia, depressão, uso de substâncias psicoativas). No entanto, o MHLq supre tais lacunas, ao propor uma avaliação a partir de uma perspectiva abrangente do construto, em vez de focar em um número restrito de transtornos mentais ou dimensões específicas destes^(22)^.

Em relação à s etapas de equivalência idiomática e semântica de itens, verificou-se que o processo de traduação inicial do instrumento original, feito por profissionais com domínio da língua portuguesa (semântica), foi importante para adequação dos termos. É importante considerar o perfil dos tradutores para obtenação de uma boa equivalência conceitual, de itens e semântica entre a versão original e traduzida^(23)^.

Na etapa de equivalência idiomática e semântica, outro aspecto a destacar é que, na retrotraduação, além de os tradutores serem bilíngues e com formação nas áreas de letras, linguística e saúde, essa etapa foi realizada de forma cega em relação à etapa de traduação inicial. Além da obtenação das versões retrotraduzidas e do consenso entre ambos, houve a necessidade de avaliação de um terceiro profissional com conhecimento do português de Portugal e Brasil e também com conhecimento na área de estudo do instrumento, aspecto destacado na literatura^(10)^. Ademais, a etapa de retrotraduação do instrumento é considerada um processo de avaliação da validade de conteúdo, na medida em que é analisado se a versão traduzida reflete com precisão o conteúdo do instrumento original.

A composiação multidisciplinar do comitê de especialistas, que envolveu profissionais da assistência, pesquisadores das áreas da saúde mental, juventude e psicometria, uma das tradutoras iniciais e um integrante de origem estrangeira e bilíngue no início do estudo, foi fundamental para o processo de adaptação transcultural.

O pré-teste, compreendido como a última etapa do processo de adaptação transcultural, cujo objetivo é analisar a compreensão da população-alvo e a aplicabilidade do instrumento à realidade local, é comum na literatura^(24,25)^. Essa etapa também foi considerada uma análise da validade de conteúdo do instrumento adaptado, visto que permitiu aos pesquisadores confirmar se este era aplicável à outra realidade.

Anteriormente^(26)^, utilizou-se um instrumento de avaliação ao final da entrevista. No presente estudo, optou-se pelo *cognitive debriefing* ^(27)^. A cada pergunta, oferecia-se a oportunidade ao respondente de dizer abertamente o que entendeu sobre a pergunta, com a finalidade de evitar erros sistemáticos de compreensão, que, ao serem detectados, eram ajustados com o comitê de especialistas, estratégia essencial para superar limitações impostas pelo ambiente virtual.

Os resultados deste estudo mostraram-se positivos, posto que as equivalências conceitual e de itens apresentaram-se relevantes (98,9%) e viáveis (97,2%) para o contexto brasileiro, haja vista que os valores de IVC tiveram resultados superiores aos parâmetros de aprovação indicados na literatura^(19,20)^. As equivalências semântica, idiomática e operacional foram essenciais para a adequação do instrumento à realidade brasileira. Os itens mostraram-se de fácil compreensão pela população-alvo.

### Limitações do estudo

Aponta-se o fato de que os dados foram coletados durante a pandemia de COVID-19, o que pode ter impactado os resultados, uma vez que a conscientização sobre problemas relacionados à saúde mental geralmente aumentou durante esse período. Devido a este fato, o pré-teste ocorreu totalmente virtual, e para o manejo de plataformas *online*, exige-se um letramento mínimo, aspecto que pode ter excluído participantes com baixos níveis educacionais, além da própria condiação de acesso à s tecnologias digitais.

### Contribuições para as áreas da enfermagem, saúde e políticas públicas

Os resultados deste estudo são oportunos e contribuem para atender aos ODS da ONU, em especial nos tópicos 3 e 4^(27)^. Relativamente à realidade brasileira, subsidiarão ações e estratégias que contribuam para o fortalecimento de sociedades com maiores níveis de letramento em saúde mental, bem com o para o avanço das políticas públicas, sobretudo voltadas para adolescentes e jovens^(28)^.

Além disso, será uma ferramenta disponível para a pesquisa epidemiológica em saúde mental no Brasil, permitindo a comparabilidade entre estudos nacionais e internacionais, contribuindo para o avanço científico relacionado aos problemas de saúde mental na população.

O enfermeiro, conhecedor da adolescência enquanto processo de transiação, detém um papel essencial na análise de todos os determinantes do letramento em saúde e no desenvolvimento de programas de intervenação no sentido da prevenação e promoação da saúde mental em idades jovens. Esses profissionais têm a oportunidade de liderar equipes multidisciplinares no uso desses programas. Assim, poderão utilizar e orientar o uso do referido instrumento em equipes de cuidados primários e/ou especializados em saúde mental, constituindo-se como uma ferramenta de apoio e qualificação do trabalho no à ¢mbito da saúde pública^(29-34)^.

## CONSIDERAÇÕES FINAIS

A adaptação e validação de conteúdo do MHLq para o Brasil necessitaram de ajustes importantes para conferir a equivalência de itens ao MHLq. O MHLq apresenta equivalência conceitual porque envolve o construto de interesse em suas diferentes dimensões. Apresenta equivalência semântica conforme processo de avaliação de significado conotativo e referencial. O MHLq atingiu equivalência operacional devido ao seu formato de apresentação e aplicação. A versão brasileira requer, ainda, o cumprimento das etapas de avaliação de equivalência de mensuração, como confiabilidade e validade da estrutura dimensional do instrumento.

## Data Availability

Os dados de pesquisa só estão disponíveis mediante solicitação.
